# De Novo Calculation of the Charge Carrier Mobility in Amorphous Small Molecule Organic Semiconductors

**DOI:** 10.3389/fchem.2021.801589

**Published:** 2021-12-24

**Authors:** Simon Kaiser, Tobias Neumann, Franz Symalla, Tobias Schlöder, Artem Fediai, Pascal Friederich, Wolfgang Wenzel

**Affiliations:** ^1^ Institute of Nanotechnology, Karlsruhe Institute of Technology (KIT), Karlsruhe, Germany; ^2^ Nanomatch GmbH, Karlsruhe, Germany; ^3^ Institute of Theoretical Informatics, Karlsruhe Institute of Technology (KIT), Karlsruhe, Germany

**Keywords:** organic semiconductor, KMC, *de novo*, mobility, multiscale workflow

## Abstract

Organic semiconductors (OSC) are key components in applications such as organic photovoltaics, organic sensors, transistors and organic light emitting diodes (OLED). OSC devices, especially OLEDs, often consist of multiple layers comprising one or more species of organic molecules. The unique properties of each molecular species and their interaction determine charge transport in OSCs—a key factor for device performance. The small charge carrier mobility of OSCs compared to inorganic semiconductors remains a major limitation of OSC device performance. Virtual design can support experimental R&D towards accelerated R&D of OSC compounds with improved charge transport. Here we benchmark a *de novo* multiscale workflow to compute the charge carrier mobility solely on the basis of the molecular structure: We generate virtual models of OSC thin films with atomistic resolution, compute the electronic structure of molecules in the thin films using a quantum embedding procedure and simulate charge transport with kinetic Monte-Carlo protocol. We show that for 15 common amorphous OSC the computed zero-field and field-dependent mobility are in good agreement with experimental data, proving this approach to be an effective virtual design tool for OSC materials and devices.

## Introduction

The discovery of electroluminescence in organic semiconductors (OSC) by Tang and VanSlyke (1987) triggered an intense research in OSC, leading to their use in a wide range of very thin and potentially printable applications, such as organic solar cells (OPV) (Baran et al., 2018) and organic field-effect transistors (OFET) (Wang et al., 2018) and their common use in organic light emitting diodes (OLEDs) (Fröbel et al., 2018). Over the past 3 decades, OLEDs have evolved significantly from the crude two-layer material used by Tang and VanSlyke into intricate multilayer thin-film devices and are commonly used in displays (Fröbel et al., 2018) and lighting applications (Tsujimura, 2017). Each of these layers consist of one or multiple species of molecules, forming thin amorphous films. One limiting property of these amorphous OSCs is the charge carrier mobility, which is several orders of magnitude below values for inorganic semiconductors (Friederich et al., 2016). The fundamental reason for this shortcoming is the localization of electrons on individual molecules in the amorphous films due to disorder of molecular electronic states. In the past 3 decades, intense research into developing materials with improved properties, including increased mobility. This research, however, was mainly lead by chemical intuition and experimental synthesis and device fabrication, limiting the progress in the exploration of the vast molecular space. On the other hand, improvements in theoretical and computational methods in OSCs have lead to these methods becoming an indispensable tool in material development and characterisation. A further increase of the accuracy could boost virtual design, enabling researchers to focus experimental efforts in the material development process to promising candidates identified in computer simulations (Friederich et al., 2017).

Seminal work by Bässler (1993) quantitatively showed the crucial dependence of charge carrier mobility on the inter-molecular disorder of electronic states. Assuming a Gaussian distribution of electronic states with width *σ*, he found a strong dependence of the charge carrier mobility on *σ* of

$$\mu \propto exp\left(-{\left(\frac{2}{3}\frac{\sigma }{k_{\text{B}}T}\right)}^{2}\right).$$ (1)

Consequently, any *de novo* mobility model requires information about the distribution of the polaron energy levels in the unique molecular environment in the amorphous film. While these quantities can only be accurately calculated in very small systems, the percolative nature of charge transport in OSCs demands a description on the O(100nm) scale (Massé et al., 2014).

This problem of scales can be solved using a multiscale simulation approach, where molecular properties computed using quantum chemistry methods are mapped to the device scale in multiple simulation steps (Kordt et al., 2015; Symalla et al., 2016; Symalla et al., 2018; Symalla et al., 2019; Symalla et al., 2020a; Symalla et al., 2020b). In these workflows, digital twins of OSC amorphous films are constructed and material specific properties determining charge carrier mobility, such as energy disorder, are computed.

In prior work we used a closed analytic expression to calculate the charge carrier mobility (Friederich et al., 2016). This expression is derived by averaging over all intermolecular hops, assuming homogeneous charge transport and thus neglecting percolative effects (Rodin et al., 2015). We used this approach to calculate the hole mobility for a set of materials commonly used in OSCs (Friederich et al., 2016) and reached about an order of magnitude agreement with experimental data. However, this approach is limited to pristine systems with a Gaussian distribution of states and does not take into account charge carrier interaction. Other work used a coarse grained approach (Baumeier et al., 2012; Kordt et al., 2015; Massé et al., 2016), mesoscale representations of organic thin films to model charge transport. Solving the Pauli Master-equation for charge carriers in the system allows the calculation of the mobility, takes electron-electron interaction into account on a mean-field level and allows for arbitrary distributions of states. This method has been effectively applied to study behavior of charge carrier mobility in realistic OSC materials (Massé et al., 2016; Kotadiya et al., 2018). This approach does not take into account many-particle effects beyond the mean-field, limiting its accuracy (van der Holst et al., 2011; Liu et al., 2017). Here we apply an approach, where the charge transport at the mesoscopic scale is computed using a kinetic Monte-Carlo approach (van der Holst et al., 2011; Kordt et al., 2015; Yavuz et al., 2015; Symalla et al., 2016; Liu et al., 2017), based entirely on *ab initio* input. This method is numerically demanding, but can be extended to simulate mixed films, interfaces or devices with ohmic injection at small applied voltages or medium to large charge carrier concentrations (van der Holst et al., 2011; Symalla et al., 2016; Liu et al., 2017).

Apart from the choice of the model to solve the transport problem, the prediction quality of the computed mobility strongly depends on the accuracy of the computed material properties, like disorder, which are highly sensitive to both details of the protocol to compute electronic structure of molecules in thin films and the underlying morphology. In contrast to our previous approach we use here a protocol mimicking physical vapour deposition to generate morphologies with atomistic resolution (Neumann et al., 2013) which is then the basis of calculations (Friederich et al., 2014) using a quantum embedding scheme. As the systems which are generated in this way are too small to be used in the kMC transport model (Massé et al., 2014) we use a stochastic extension scheme (Baumeier et al., 2012; Symalla et al., 2016). This scheme has been used to investigate charge and energy transport in emissive layers (Symalla et al., 2020a; Symalla et al., 2020b), doped injection layers (Symalla et al., 2020a), single-carrier devices (Kaiser et al., 2021) and OLEDs (Symalla et al., 2018; Symalla et al., 2019) *de novo*.

Clearly the complex interplay of these methods calls for a thorough benchmark of the method. This benchmark is all the more important, because bottom-up device calculations based on *ab initio* input cannot be performed with continuum models, but are—ideally—conducted by kMC calculations. Such calculations would fail if the electronic structure of the individual materials are not correctly represented. To ensure the quality of our single materials model, we apply this workflow to compute both zero-field and field-dependent mobility without the need for experimental input for a wide range of materials commonly applied in electron or hole transport layers or as host materials in emission layers. With only the molecular structure as input, the computed zero-field and field-dependent mobilities show a significantly improved agreement between simulation and experiment.

## Methods

The multiscale workflow mentioned in the introduction consists of five basic steps, which are detailed below: First, density-functional theory (DFT) (Kohn and Sham, 1965) is used to optimize the geometry and compute partial charges via an electrostatic potential (ESP) fit (Singh and Kollman, 1984) for single molecules in vacuum. Subsequently, the optimized molecular structures as input, morphologies with atomistic resolution are generated using a Monte-Carlo based protocol mimicking physical vapor deposition (Neumann et al., 2013). Using the QuantumPatch method (Friederich et al., 2014), we calculate the energy disorder, electronic couplings and reorganization energies in these thin film representations by self-consistently equilibrating the charge densities of a subset of molecules in their respective unique environment for a limited system size of $O\left(1000\right)$ molecules. To bridge the scales of atomistic resolution and device-level, we use the distance distribution in the morphology to stochastically generate thin film morphologies of several 10 000 to 100 000 sites and draw electronic couplings, site and reorganization energies from the individual distributions analyzed with the QuantumPatch method energies (Baumeier et al., 2012; Symalla et al., 2016). Based on these expanded morphologies we simulate charge transport in OSC thin films using the kMC protocol lightforge (LF) (Symalla et al., 2016). In LF, charge carriers are modeled as single entities that hop on neighbouring sites with rates derived from Marcus theory (Marcus, 1956). These charge transport simulations allow the computation of field-dependent and zero-field mobility, taking into account percolation and many-body effects (van der Holst et al., 2011; Liu et al., 2017). All DFT calculations are performed using the Turbomole (Ahlrichs et al., 1989) DFT package.

### Morphology Generation

To obtain amorphous thin-film morphologies for each molecule, the DEPOSIT (Neumann et al., 2013) protocol is used to simulate physical vapour deposition. Molecules are added to the simulation box one at a time, scanning the morphology surface using a MC based basin hopping with simulated annealing (SA). Intermolecular interactions during the deposition process are modeled using Lennard-Jones potentials (parameters listed in Supplementary Table S1) and Coulomb potentials based on the ESP charges derived as described above. To model different molecular configurations, rotations of dihedrals around single bonds are performed, bond distances and angles are kept fixed during deposition. The energy of the various configurations is computed using molecule-specific intramolecular force-fields derived by step-wise rotation of dihedral angles of single molecules in vacuum and computing DFT energies of each configuration using the B3LYP (Stephens et al., 1994) functional and def2-SVP (Weigend and Ahlrichs, 2005) basis set. To improve the sampling, multiple SA cycles are run in parallel, with one molecule selected based on the Metropolis-criterion (Hastings, 1970). After deposition, each molecule is kept fixed to achieve linear scaling of computing time. To obtain representative atomistic models of the amorphous thin-film for each material, 2000 molecules are deposited into a simulation box of 90Å x 90Å x 360Å with 32 parallel SA cycles starting at an artificially high temperature (4000K) and cooling to room temperature (300K) in 130 000 MC steps, providing sufficient sampling for the molecules studied here (Friederich et al., 2018).

### Electronic Structure Calculation

Orbital energy differences Δ*E*_*ij*_, electronic couplings *J*_*ij*_ and reorganization energies *λ* are calculated for a subset of molecules in their converged unique electrostatic environments in the thin-film morphologies using the QuantumPatch method (Friederich et al., 2014), which self-consistently equilibrates the charge densities of the molecules in the film.

Using the equilibrated orbital energies of the innermost 200 molecules, the energy disorder $\sigma =\frac{1}{\sqrt{2}}\sigma \left(\Delta E_{ij}\right)$ is calculated from the standard deviation of energy differences Δ*E*_*ij*_ of all pairs of neighbouring molecules *i* and *j*. Energy evaluations are performed using the B3LYP functional and def2-SVP basis set.

In the converged system, the electronic couplings *J*_*ij*_ of a molecule *i* to its respective neighbour *j* are calculated using the Löwdin orthogonalization procedure (Löwdin, 1950) for the highest occupied molecular orbital (HOMO) and lowest unoccupied molecular orbital (LUMO). Electronic couplings are calculated for pairs of the innermost 150 molecules with an atom-atom distance of less than 7Å. Dimer DFT calculations are performed using the BP86 (Perdew, 1986; Becke, 1988) functional and def2-SVP basis set.

Reorganization energies *λ*_*i*_ are calculated based on Nelsen’s four point procedure (Nelsen et al., 1987) for eleven core molecules in their unique environment. The geometry of the charged and uncharged molecules are optimized within constraints imposed by their environments. Constraints are implemented by placing effective core potentials (ECP) at the position of atoms of neighbouring molecules. DFT calculations are performed using the B3LYP functional and def2-SVP basis set.

### Structure Expansion

Accurate modelling of charge transport in these amorphous OSCs requires system sizes of several 10 nm in each direction (Massé et al., 2014). The deposited thin films are therefore expanded into an amorphous structure of arbitrary size using an extension of the dominance competition model of Baumeier et al. (2012), which we presented in prior work (Symalla et al., 2016).

In this expanded amorphous structure, on-site energies are drawn following the Gaussian distributions of Δ*E*_*ij*_ obtained from the electronic structure calculations. Electronic coupling elements *J*_*ij*_ for site *i* and one of its neighbours *j* are drawn from the microscopic distribution $J\left(r\right)$ within the small interval d*r* around the distance *r*_*ij*_ between both sites. Reorganization energies are taken to follow a Gaussian distribution.

### Charge Transport Simulation

Field-dependent charge transport in the OSC is simulated with the lightforge (LF) package (Symalla et al., 2016). Charge transport is modelled as hopping transport from site *i* to any of it’s neighbours *f* with a rate

$$k_{if}=\frac{2\pi }{\hbar }|J_{if}{|}^{2}\frac{1}{\sqrt{4\pi \lambda k_{\text{B}}T}}\exp\left(-\frac{{\left(\lambda +\Delta E_{if}\right)}^{2}}{4\lambda k_{\text{B}}T}\right)$$ (2)

based on Marcus-theory (Marcus, 1956), where *λ* is the reorganization energy, *T* the temperature, *J*_*if*_ the transfer integral and Δ*E*_*if*_ the energy difference between this charge occupying site *i* and site *f* due to the energy disorder of the amorphous system, the applied field and the dynamic electrostatic potential of all other charges in the system. *J*_*if*_ contains both the direct electronic coupling of sites *i* and *f* and the superexchange coupling via any of the *N* neighbouring sites *j* using first-order perturbation theory

$$J_{if}\approx J_{if,0}+\overset{N}{\underset{j\neq i;j\neq f}{\sum }}\frac{J_{ij,0}J_{jf,0}}{E_{\text{virt}}-E_{\text{T}}}$$ (3)

where *J*_*if*,0_ is the direct electronic coupling of sites *i* and *f*, *E*_virt_ is the energy of the system in its virtual state with the charge occupying site *j* and *E*_T_ the transition state energy (Symalla et al., 2016). Coulomb interactions with the nearest periodic copy of all other charges are treated explicitly, resulting in an effective cutoff of half the system size.

To model mobility measurements in the bulk of an OSC, charge transport is simulated with a charge carrier density of 1 × 10^–3^ per site in an amorphous system of 40 nm × 40 nm × 40 nm with periodic boundary conditions in *x*-, *y*- and *z*-direction. To account for stochastics in morphology expansion and site-energy distribution, we sample 10 different configurations per applied field. Connectivity of a given pair of sites with distance *d* is determined by the probability of a pair of molecules with a center of mass distance *d* having a nearest atom distance of less than 7Å. Hopping transport is possible between all connected pairs with both direct and superexchange coupling taken into account. Convergence is reached if the current density is constant over two thirds of the simulation.

## Results

**FIGURE 1:**
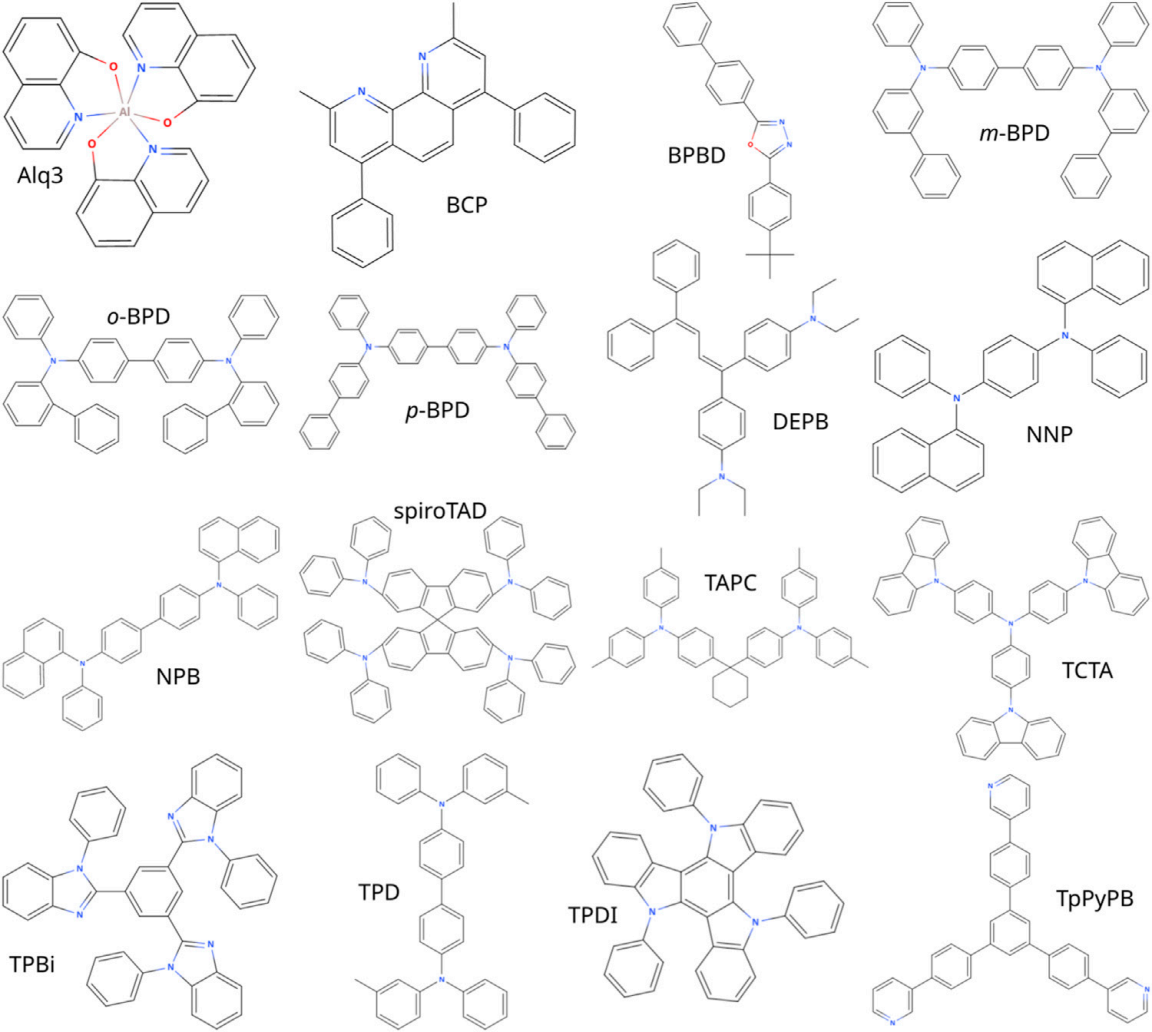
Structures of the molecules characterized in this work.

We apply the presented workflow to calculate the mobilities of the materials shown in Figure 1, namely the hole-transport materials *N*, *N*′-di (biphenyl-3-yl)-*N*, *N*′-diphenyl-[1,1’-biphenyl]-4,4’-diamine (*m*-BPD), *N*, *N*′-di (biphenyl-2-yl)-*N*, *N*′-diphenyl-[1,1’-bi phenyl]-4,4’-diamine (*o*-BPD), *N*, *N*′-di (biphenyl-4-yl)-*N*, *N*′-diphenyl-[1,1’-biphenyl]-4,4’-diamine (*p*-BPD), 1,1-bis-(4,4’-diethyl amino phenyl)-4,4-di phenyl-1,3-buta diene (DEPB), *N*, *N*′-bis(1-naphthalen yl)-*N*, *N*′-di phenyl-4,4’-phenyl di amine (NNP), *N*, *N*′-di (1-naph thyl)-*N*, *N*′-di phenyl-(1,1’-biphenyl)-4,4’-diamine (NPB), 2,2’,7,7’-tetra kis (*N*, *N*-diphenyl amine)-9,9’-spiro bifluorene (spiroTAD), di-[4-(*N*, *N*-ditolyl-amino)phenyl]cyclo hexane (TAPC), 4,4’,4″-tris(*N*-carbazolyl)tri phenyl amine (TCTA), *N*, *N*′-diphenyl-N,N’-bis-(3-methyl phenylene)-1,1’-diphenyl-4, 4’-diamine (TPD), and 5,10,15-triphenyl-5H-diindolo [3,2-a:3’,2’-c]carbazole (TPDI), electron-transport materials 2,9-di methyl-4,7-diphenyl-1,10-phenanthrolin (BCP), 2-(4-biphenylyl)-5-(4-tert-butyl phenyl)-1,3,4-oxa di azole (BPBD), 2,2’,2”-(1,3,5-benzine tri yl)-tris(1-phenyl-1-H-benzimid azole) (TPBi) and 1,3,5-tri (p-pyrid-3-yl-phenyl)benzene (TpPyPB) and both hole- and electron transport material tris(8-hydroxy quinoline)aluminum (Alq3) at low fields. From these field-dependent mobilities we extrapolate the zero-field mobilities.

To compare the field dependence of computed and measured mobilities, experimental works featuring field-dependent mobilities were considered as references. For materials with more than one reference, mobilities measured using the time-of-flight (ToF) technique (Kepler, 1960; Kepler et al., 1995) are preferred over mobilities fitted to the current in the space-charge limited regime (SCLC) (Mott and Gurney, 1957; Murgatroyd, 1970) due to issues in fabrication, measurement and analysis reported by Blakesley et al. (2014). If multiple ToF measurements are available, we used the ones featuring the highest mobility, as we expect these to be the most well-prepared films with least impurities. Following this selection process, measured field-dependent mobilities reported by Naka et al. (1999) (Alq3_p_), Liu et al. (2010) (Alq3_n_ and BCP), Hung et al. (2006) (TPBi), Kawabe and Abe (2002) (BPBD), Mori et al. (1993) (DEPB and TPD), Okumoto et al. (2000) (*m*-, *o*- and *p*-BPD), Borsenberger and Shi (1995) (NNP), Bach et al. (2000) (spiroTAD), Noh et al. (2009) (TCTA), Naka et al. (2000) (NPB), Su et al. (2008) (TpPyPB), Huh et al. (2013) (TPDI) and Borsenberger et al. (1991) (TAPC) are taken as reference for simulated mobilities. The mobility of BPBD was fitted to the emission response from a bilayer TPD–BPBD stack, the mobility of TPDI was fitted to the SCLC. All other mobilities were measured in pure devices using the ToF technique. Mobilities predicted by theoretical multiscale models reported by Friederich et al. (2016) (Alq3_p_, DEPB, *m*-BPD, NNP, NPB, TPD), Kordt et al. (2015) (BCP and NPB, Massé et al. (2016) (TCTA and NPB), Kotadiya et al. (2018) (spiroTAD, TCTA and NPB), Evans et al. (2016) (*m*-, *o*- and *p*-BPD, TCTA, NPB and TPD), Fuchs et al. (2012) (Alq3_p_ and Alq3_n_; field-dependent) and Aydin and Yavuz (2021) (NPB, TPD and TAPC; field-dependent) are used to assess the presented workflow. Where required, zero-field mobilities are extrapolated from field-dependent mobilities.

**FIGURE 2:**
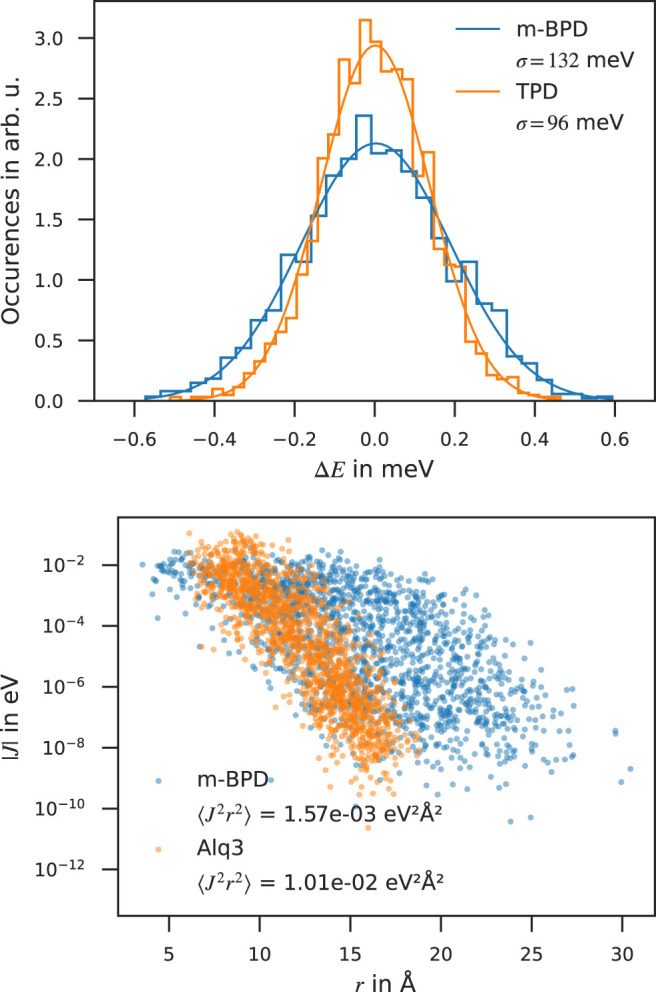
Electronic properties computed with the QuantumPatch method. Top panel: Distribution of HOMO energy differences in *m*-BPD and TPD along with the fitted Gaussian distribution yielding the energetic disorder *σ*. Bottom panel: Distribution of electronic couplings of *m*-BPD and Alq3.

The disorder width *σ*, ⟨*J* ^2^ *r* ^2^⟩ and *λ* are measures for the disorder, electronic couplings and the reorganization energy, respectively, which mainly determine the transport properties. Table 1 lists these values for each molecule calculated with our multiscale workflow along with zero-field mobilities extrapolated from field-dependent mobilities simulated with our kMC model and reported from experiment and theory. The distribution of energy differences Δ*E*_*ij*_, depicted in the left panel of Figure 2 along with the fitted Gaussian distribution of width *σ* for two molecules, namely *m*-BPD and TPD, strongly influences the mobility. With comparable ⟨*J* ^2^ *r* ^2^⟩ and *λ*, but a 38% higher *σ*, the simulated zero-field mobility of *m*-BPD lies a factor of 65 below that of TPD. The distribution of electronic couplings for two molecules, namely *m*-BPD and Alq3, are shown in the right panel of Figure 2 for comparison. Alq3, without any dihedral angles, is rigid in our deposition scheme, resulting in densely packed structures and a narrow distribution of *J*_*ij*_ which decays fast with the neighbour distance, while more extended and flexible molecules, e. g., *m*-BPD, show a broader distribution of *J*_*ij*_s which decay slower with the neighbour distance. These differences are reflected in the values of ⟨*J* ^2^ *r* ^2^⟩ which for these molecules differ by almost one order of magnitude between both molecules. With the electronic couplings *J*_*ij*_ only entering as a prefactor in the Marcus-rate (Eq. 2) and *σ* in the exponent, differences in *σ* have a far larger impact on the mobility than differences in electronic couplings *J*_*ij*_, as is evident e. g., by comparing Alq3 and TPBi electron mobilities or both *p*-BPD and TAPC mobilities ranking among the highest three despite their average electronic couplings ranking among the lowest. The environment inhibits the full structure relaxation of a molecule after charge transfer, leading to *λ* well between in-vacuo calculations with calculations in the solid phase with constrained dihedral angles to inhibit large-scale conformational changes (frozen dihedral approximation) (Friederich et al., 2016).

**TABLE 1:** Electronic properties, namely energetic disorder, mean electronic coupling and reorganization energy, computed with the QuantumPatch method and zero-field mobilities simulated with our kMC model. Experimental zero-fields mobilities reported in literature are listed for comparison.

Molecule	*σ*/meV	$\left\langle J^{2}r^{2}\right\rangle /{eV}^{2}{\overset{{}^{\circ}}{A}}^{2}$	*λ*/meV	*μ*_0_/cm^2^V^−1^s^−1^	
				kMC	Experiment
Alq3_p_	199	1.0 × 10^–02^	195	2.6 × 10^–09^	5.7 × 10^–10^ ^a^
Alq3_n_	182	8.6 × 10^–03^	215	1.7 × 10^–07^	7.4 × 10^–08^ ^b^
TPBi_n_	157	2.5 × 10^–03^	317	4.3 × 10^–07^	4.7 × 10^–07^ ^c^
BPBD_n_	182	5.2 × 10^–03^	291	3.6 × 10^–06^	1.3 × 10^–06^ ^d^
DEPB_p_	133	2.4 × 10^–03^	316	6.0 × 10^–06^	1.2 × 10^–05^ ^e^
*m*-BPD_p_	132	1.6 × 10^–03^	210	8.8 × 10^–06^	1.5 × 10^–05^ ^f^
BCP_n_	136	3.2 × 10^–03^	314	1.4 × 10^–05^	3.1 × 10^–05^ ^b^
NNP_p_	124	1.6 × 10^–03^	281	1.2 × 10^–05^	3.9 × 10^–05^ ^g^
spiroTAD_p_	105	1.7 × 10^–03^	139	8.7 × 10^–05^	1.5 × 10^–04^ ^h^
TCTA_p_	107	1.7 × 10^–03^	206	1.3 × 10^–04^	1.8 × 10^–04^ ^i^
NPB_p_	104	1.4 × 10^–03^	205	1.8 × 10^–04^	2.9 × 10^–04^ ^j^
*o*-BPD_p_	96	1.8 × 10^–03^	213	3.2 × 10^–04^	4.4 × 10^–04^ ^f^
TpPyPB_n_	123	6.4 × 10^–03^	200	3.0 × 10^–04^	6.5 × 10^–04^ ^k^
TPD_p_	93	1.7 × 10^–03^	208	7.9 × 10^–04^	6.7 × 10^–04^ ^e^
*p*-BPD_p_	94	1.3 × 10^–03^	173	7.0 × 10^–04^	7.2 × 10^–04^ ^f^
TPDI_p_	82	4.8 × 10^–03^	145	1.0 × 10^–03^	2.3 × 10^–03^ ^L^
TAPC_p_	74	1.4 × 10^–03^	89	4.6 × 10^–03^	5.6 × 10^–03^ ^m^

a Naka et al. (1999).

b Liu et al. (2010).

c Hung et al. (2006).

d Kawabe and Abe (2002).

e Mori et al. (1993).

f Okumoto et al. (2000).

g Borsenberger and Shi (1995).

h Bach et al. (2000).

i Noh et al. (2009).

j Naka et al. (2000).

k Su et al. (2008).

l Huh et al. (2013).

m Borsenberger et al. (1991).

**FIGURE 3:**
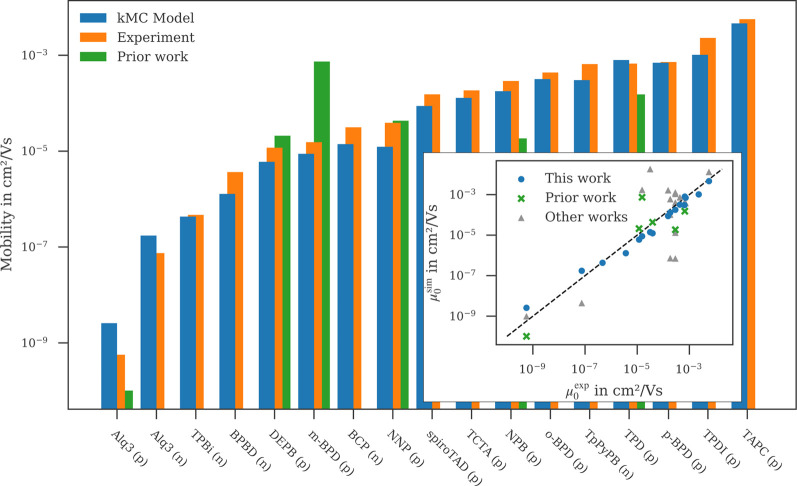
Zero-field mobilities of both hole- and electron transport materials calculated with the presented kMC model compared to experimental data (see text) and prior work (Friederich et al., 2016) where available. Inset: Comparison of zero-field mobilities computed in this work (blue circle), prior work (Friederich et al., 2016) (green cross) and reported in literature (see text) (grey triangle) with experimental values reported in literature (see text). Linear relationship between simulation and experiment is shown by the black dashed line.

Figure 3 shows the zero-field mobility obtained with the kMC model compared to data from experiment and prior work (Friederich et al., 2016). As can be seen, the kMC zero-field mobilities are in very good agreement with the experimental data. With the exception of Alq3, all mobilities lie within 50% of the experimental data and fully reproduce the experimental trends. Going beyond the Gaussian disorder model to a more accurate description of the local distribution of energy levels, as described below, not only recovers the order of mobility for BCP and NNP, but also reproduces experimental mobility within a few percent for both materials. The systematic overestimation of Alq3 hole and electron mobility can be attributed to our approximation that the Al-complex stays rigid during deposition, which leads to an underestimation of the disorder. The zero-field mobilities obtained in prior work, lie within two orders of magnitude above and one order of magnitude below the experimental zero-field mobilities with a slight trend of overestimating the experimental data.

**FIGURE 4:**
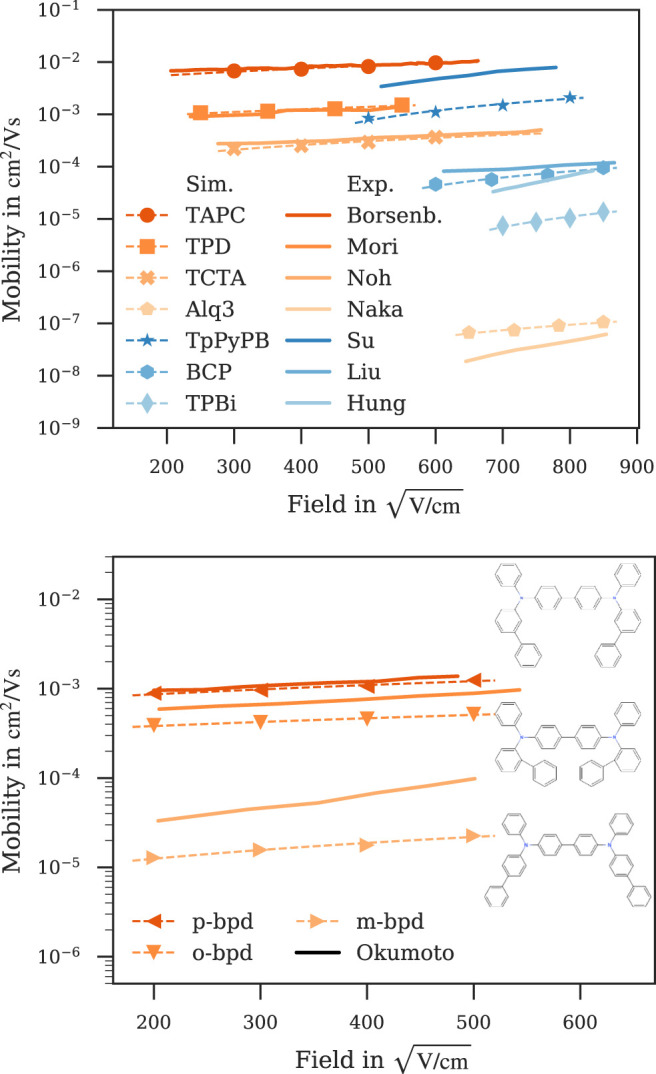
Computed field-dependent mobilities for a subset of materials compared to experimental data reported in literature. Both the magnitude and field-dependence of the simulated mobilities agree well with experimental data. Top panel: Hole mobilities (orange) of TAPC (Borsenberger et al., 1991), TPD (Mori et al., 1993), TCTA (Noh et al., 2009) and Alq3 (Naka et al., 1999) and electron mobilities (blue) of TpPyPB (Su et al., 2008), BCP (Liu et al., 2010) and TPBi (Hung et al., 2006). Bottom panel: The presented workflow captures the difference in mobility due to the small modifications in the *m*-, *o*- and *p*-BPD isomers (Okumoto et al., 2000; inset shows their respective structures). Dashed lines are fitted to Poole-Frenkel behaviour $\ln\left(\mu \right)\propto \gamma \sqrt{E}$ , simulation errors are of the order of symbol size.

While zero-field mobility is a readily accessible descriptor when optimizing or searching for new candidate molecules (Friederich et al., 2017), the performance of the material in a device is determined by the charge carrier mobility at fields relevant for the specific application. Figure 4 shows field-dependent mobilities computed with our kMC model for a subset of materials (see Figure 6; Supplementary Figure S3 for the other materials) and experimental mobilities reported in literature (Borsenberger et al., 1991; Naka et al., 1999; Naka et al., 2000; Okumoto et al., 2000; Hung et al., 2006; Su et al., 2008; Noh et al., 2009; Liu et al., 2010) for comparison. As can be seen, the field-dependence predicted by the kMC model matches the experimental data over a wide range of fields. Considering only charge carrier mobility at zero field BCP is a far better electron conductor than TPBi, while the mobilities get closer at relevant fields due to the strong field-dependence of TPBi, with the mobility of TPBi eventually surpassing that of BCP at large fields. Alq3, as a hole-transport material, shows an equally strong field-dependence, stronger than that of e. g., TAPC, TPD or TCTA, the five orders of magnitude difference in their zero-field mobilities still lead to Alq3 being the inferior hole-transport material at relevant fields. It is interesting to note that our model successfully captures differences in charge carrier mobility due to small molecular modifications as with the three isomers *m*-, *o*- and *p*-BPD (Figure 4, top panel). While the simulation slightly underestimates the field-dependence of *m*-BPD and overestimates the difference between *o*- and *p*-BPD in this field range, it captures both the general trends and individual ordering of the three isomers.

**FIGURE 5:**
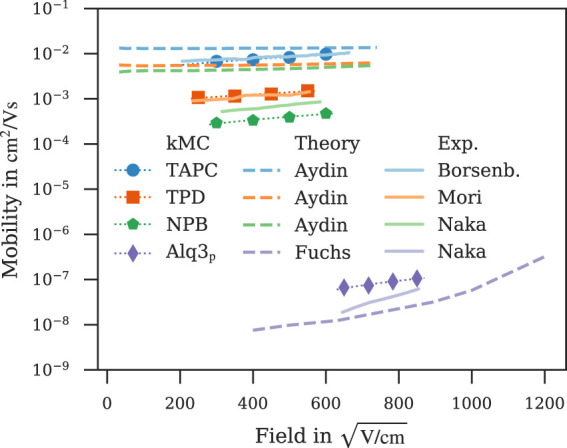
Comparison of field-dependent mobilities computed with *ab initio* multiscale models reported in this work (symbol) and literature (Fuchs et al., 2012; Aydin and Yavuz, 2021) (dashed line) with experiment (Borsenberger et al., 1991; Naka et al., 1999; Naka et al., 2000) (solid line). Dotted lines are fitted to Poole-Frenkel behaviour, simulation errors are of the order of symbol size.

Figure 5 shows a comparison of field-dependent mobilities computed with *ab initio* multiscale workflows reported here and in literature (Fuchs et al., 2012; Aydin and Yavuz, 2021) along with experimental data (Borsenberger et al., 1991; Naka et al., 1999; Naka et al., 2000). Field-dependent mobilities of TAPC, TPD and NPB simulated with our kMC model show a better agreement with experiment (Borsenberger et al., 1991; Mori et al., 1993; Naka et al., 2000) than the model by Aydin and Yavuz (2021) despite both works using a kMC model for charge-transport simulations with comparable values for energetic disorder (Supplementary Table S2). The Master-equation model by Fuchs et al. (2012) underestimates the Alq3 mobility by approximately the same factor our kMC model overestimates it.

The coarse graining approach we applied to generate mesoscale systems draws random site energies from a Gaussian density of states. This approach neglects local effects of the electronic structure, i. e., the shift of energy levels induced by the relative position and orientation of neighbouring molecules. To estimate the impact of this approximation, we compute specific energy level shifts of all molecules in the atomistic morphology based on partial charges of their neighbouring molecules derived from DFT. Expanded morphologies were then generated by periodic expansion of the deposited morphologies in *x*- and *y*-direction, resulting in cubic structures with approx. 20 000 sites. Charge carrier density was kept constant at approx. 1 × 10^–3^ per site for comparability.

**FIGURE 6:**
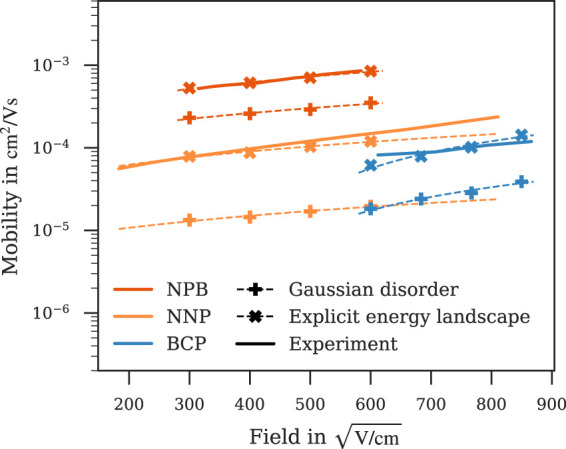
Mobilities calculated with regular Gaussian disorder (✚) or the energy landscape derived explicitly from local electrostatics (✖). Taking into account the local electrostatics improves the fit to experiment for NPB (Borsenberger and Shi, 1995), NNP (Naka et al., 2000) and BCP (Liu et al., 2010) and properly resolves the difference in NNP and BCP mobility observed in experiment. Dashed lines are fitted to Poole-Frenkel behaviour, simulation errors are of the order of symbol size.

Field-dependent mobilities obtained with both the Gaussian disorder and this energy landscape derived explicitly from local electrostatics compared with experiment are displayed in Figure 6 for NPB, NNP and BCP, for which simulations using the Gaussian disorder model lead to an underestimation of charge carrier mobility. These results indicate that taking into account the local electrostatic effects within the atomistic morphology can have a strong impact on charge carrier mobility. In the case of NPB, NNP and BCP, it lead to an improved fit to experiment. Notably, a similar improvement was not observed for all materials. The advanced approach to include local effects in the electronic structure limits the system size to the size of the atomistic morphologies. This statistical limitation may lead to a large fluctuation of mobility between deposited samples due to percolation, and is therefore no general substitute to the Gaussian disorder on expanded morphologies. The mobilities displayed in Figure 6, however, indicate that including local electrostatic effects in the energy landscape may improve prediction quality of charge carrier simulations.

## Discussion

Low charge carrier mobility in OSC materials limits the potential of OSC devices including OLEDs. Computational methods can speed up characterization of new material candidates, helping explore the vast molecular space and boost virtual design. A computational method for mobility prediction requires an accurate representation of the thin-film morphology and material properties, e. g., the disorder of polaron energies. Computation of these material properties in turn requires precise quantum-chemistry methods that take into account the unique environment of each molecule in the amorphous morphology.

We present here an *ab initio* multiscale workflow to compute material properties and simulate charge transport and benchmark computed mobilities in 15 organic thin films. To this end, we generate atomistic models of amorphous thin films with molecular mechanics calculations using customized force-fields derived from quantum-mechanics (Neumann et al., 2013). We then calculate the electronic structure of molecules in these thin film morphologies using a quantum embedding method (Friederich et al., 2014) and transfer material properties, specifically molecular energy levels, electronic couplings and site distributions, *via* an extension scheme to kinetic Monte-Carlo simulations to compute the charge transport properties. With this parameter free approach we achieve good agreement to experimental data for computed zero-field and field-dependent mobilities of a wide range of molecules frequently used in OLED stacks.

This multiscale model to predict charge carrier mobility can aid experimental R&D towards the design of efficient OLED materials and devices in three ways: First, without the need to parametrize this model, e. g., with experiment, computation of charge carrier mobility enables full virtual screening of materials, thereby allowing to focus experimental efforts to most promising candidates. Second, by bridging the gap between fundamental chemistry and mesoscopic material properties, the presented workflow can aid in gaining systematic understanding on the structure-function-relationship of molecular properties and device performance, as well as derivation of design rules for new materials. Third, this *de novo* workflow can be linked seamlessly to the continuum scale, i. e., drift-diffusion, models which are widely used in OLED development. This link between the micro- and the macroscale opens the prospect of a higher level of automation in OLED design.

In the pristine systems studied here charge transport is primarily determined by the width of the Gaussian DOS. An accurate description of a mixed system additionally requires knowledge about the position of the mean values of the Gaussian DOS, i. e., mean ionization energies and electron affinities, for the different molecular species. This additional quantum chemical challenge is addressed by recent developments for accurate predictions of ionization energies and electron affinities by Armleder et al. (2021), which could be integrated in this workflow to facilitate accurate *de novo* mobility predictions in mixed systems.

Going towards more ordered systems than the amorphous molecules studied here, the transport regime crosses over from hopping transport to band-like transport. In this crosssover regime, other methods, such as Ehrenfest or surface hopping approaches, can be employed to describe charge transport (Wang et al., 2016; Oberhofer et al., 2017; Carof et al., 2019; Wang et al., 2020).

Ultimately, approaches to include simulation of exciton dynamics in similar multiscale workflows (Symalla et al., 2018; Symalla et al., 2019) and predict device efficiency based on first principles depend on an accurate description of charge carrier balance in multilayer OLEDs, and therefore a reliable model for simulating charge transport through each layer. This work is thus a fundamental step towards full virtual design in OLED technology.

## Data Availability

The original contributions presented in the study are included in the article and Supplementary Material, further inquiries can be directed to the corresponding author.
