# Supplementary material for: De Novo Calculation of the Charge Carrier Mobility in Amorphous Small Molecule Organic Semiconductors
Source: Front Chem. 2021 Dec 24;9:801589. doi: 10.3389/fchem.2021.801589 (PMC8738089; doi:10.3389/fchem.2021.801589)
Supplement: Supplementary file 1 [file DataSheet1.PDF]

Supplementary Material

Table S1. Lennard-Jones parameters of the DEPOSIT (Neumann et al. (2013)) standard forcefield.

Atom	σ/nm	$\epsilon/\mathrm{kJ\,mol^{-1}}$
C	0.356	0.63
H	0.178	0.08
O	0.285	0.84
N	0.29	0.67
Al	0.35	0.276

Table S2. Electronic properties and field-dependent mobilities computed with *ab initio* multiscale workflows reported here and in literature.

Molecule	σ/meV	λ/meV	$\mu/\mathrm{cm^2\,V^{-1}\,s^{-1}}$	$\mu_0/\mathrm{cm^2\,V^{-1}\,s^{-1}}$	Source
$\mathrm{Alq3_p}$	199	195	6.3×10^{-8} m*	2.6×10^{-9}	SK
	201	467	1.2×10^{-8} m	9.7×10^{-10}	AF
$\mathrm{Alq3_n}$	182	215	2.3×10^{-6} h*	1.7×10^{-7}	SK
	178	501	5.5×10^{-6} h	4.4×10^{-9}	AF
$\mathrm{NPB_p}$	104	205	3.3×10^{-4} l	1.8×10^{-4}	SK
	107	286	4.4×10^{-3} l*	4.0×10^{-3}	GA
$\mathrm{TPD_p}$	96	208	9.3×10^{-4} l	5.7×10^{-4}	SK
	103	273	5.6×10^{-3} l*	5.4×10^{-3}	GA
$\mathrm{TAPC_p}$	74	89	7.3×10^{-3} l	4.6×10^{-3}	SK
	70	174	1.3×10^{-2} l*	1.3×10^{-2}	GA

[SK] This work [AF] A. Fuchs et al. (2012) [GA] G. Aydin and Yavuz (2021)
[l] at 400 $\sqrt{\mathrm{V/cm}}$ [m] at 632 $\sqrt{\mathrm{V/cm}}$ [h] at 895 $\sqrt{\mathrm{V/cm}}$ [*] interpolated

Table S3. Electronic properties and zero-field mobility computed in this work and reported in literature.

Molecule	σ/meV	$\langle J^2 r^2\rangle/\mathrm{eV}^2\,\mathrm{\AA}^2$	λ/meV	$\mu_0/\mathrm{cm}^2\,\mathrm{V}^{-1}\,\mathrm{s}^{-1}$	Source
$\mathrm{Alq3_p}$	199	1.0×10^{-2}	195	2.6×10^{-9}	SK
	224	1.0×10^{-2}	296	1.0×10^{-10}	PF
$\mathrm{Alq3_n}$	182	8.6×10^{-3}	215	1.7×10^{-7}	SK
$\mathrm{TPBi_n}$	164	2.5×10^{-3}	317	4.3×10^{-7}	SK
$\mathrm{BPBD_n}$	182	5.2×10^{-3}	291	1.3×10^{-6}	SK
$\mathrm{DEPB_p}$	133	2.4×10^{-3}	316	6.0×10^{-6}	SK
	130	1.4×10^{-3}	266	2.1×10^{-5}	PF
m-$\mathrm{BPD_p}$	132	1.6×10^{-3}	210	8.8×10^{-6}	SK
	110	1.5×10^{-3}	143	7.4×10^{-4}	PF
			300	1.7×10^{-3}	DE
$\mathrm{BCP_n}$	139	3.2×10^{-3}	314	1.4×10^{-5}	SK
				1.8×10^{-2}	PK
$\mathrm{NNP_p}$	124	1.6×10^{-3}	281	1.2×10^{-5}	SK
	135	1.6×10^{-3}	160	4.3×10^{-5}	PF
$\mathrm{spiroTAD_p}$	105	1.7×10^{-3}	139	8.7×10^{-5}	SK
	90		250	1.6×10^{-3}	NK
$\mathrm{TCTA_p}$	107	1.7×10^{-3}	206	1.3×10^{-4}	SK
	136		257	7.2×10^{-7}	AM
	112		260	1.0×10^{-4}	NK
			290	5.9×10^{-4}	DE
$\mathrm{NPB_p}$	104	1.4×10^{-3}	205	1.8×10^{-4}	SK
	130		203	6.9×10^{-7}	AM
	114			1.3×10^{-5}	PK
	144	2.0×10^{-3}	158	1.8×10^{-5}	PF
	87		310	1.1×10^{-3}	NK
			280	1.3×10^{-3}	DE
o-$\mathrm{BPD_p}$	96	1.8×10^{-3}	213	3.2×10^{-4}	SK
			310	7.2×10^{-4}	DE
$\mathrm{TpPyPB_n}$	123	6.4×10^{-3}	200	3.0×10^{-4}	SK
$\mathrm{TPD_p}$	96	1.7×10^{-3}	208	7.9×10^{-4}	SK
	129	1.6×10^{-3}	110	1.5×10^{-4}	PF
			310	8.3×10^{-4}	DE
p-$\mathrm{BPD_p}$	94	1.3×10^{-3}	173	7.0×10^{-4}	SK
			230	3.8×10^{-4}	DE
$\mathrm{TPDI_p}$	82	4.8×10^{-3}	145	1.0×10^{-3}	SK
$\mathrm{TAPC_p}$	74	1.4×10^{-3}	89	4.6×10^{-3}	SK

[SK] This work [PK] P. Kordt et al. (2015) [AM] A. Massé et al. (2016) [DE] D. Evans et al. (2016) [PF] P. Friederich et al. (2016) [NK] N. Kotadiya et al. (2018)

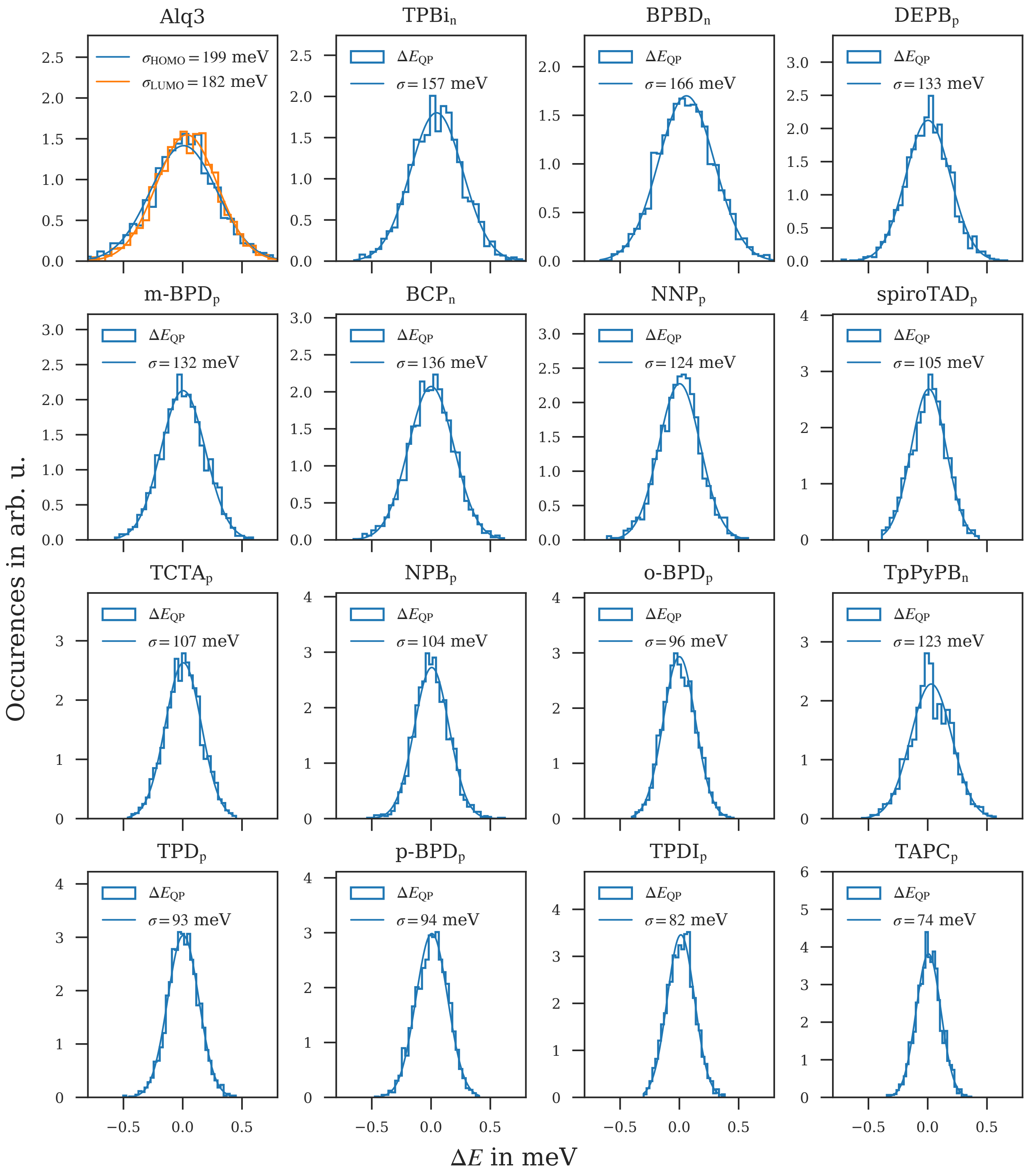

Figure S1. Distribution of ΔE computed with the QuantumPatch method (Friederich et al. (2014)), Gaussian fit and resulting disorder $\sigma = \frac{1}{\sqrt{2}}\sigma(\Delta E)$ for all molecules studied in this work.

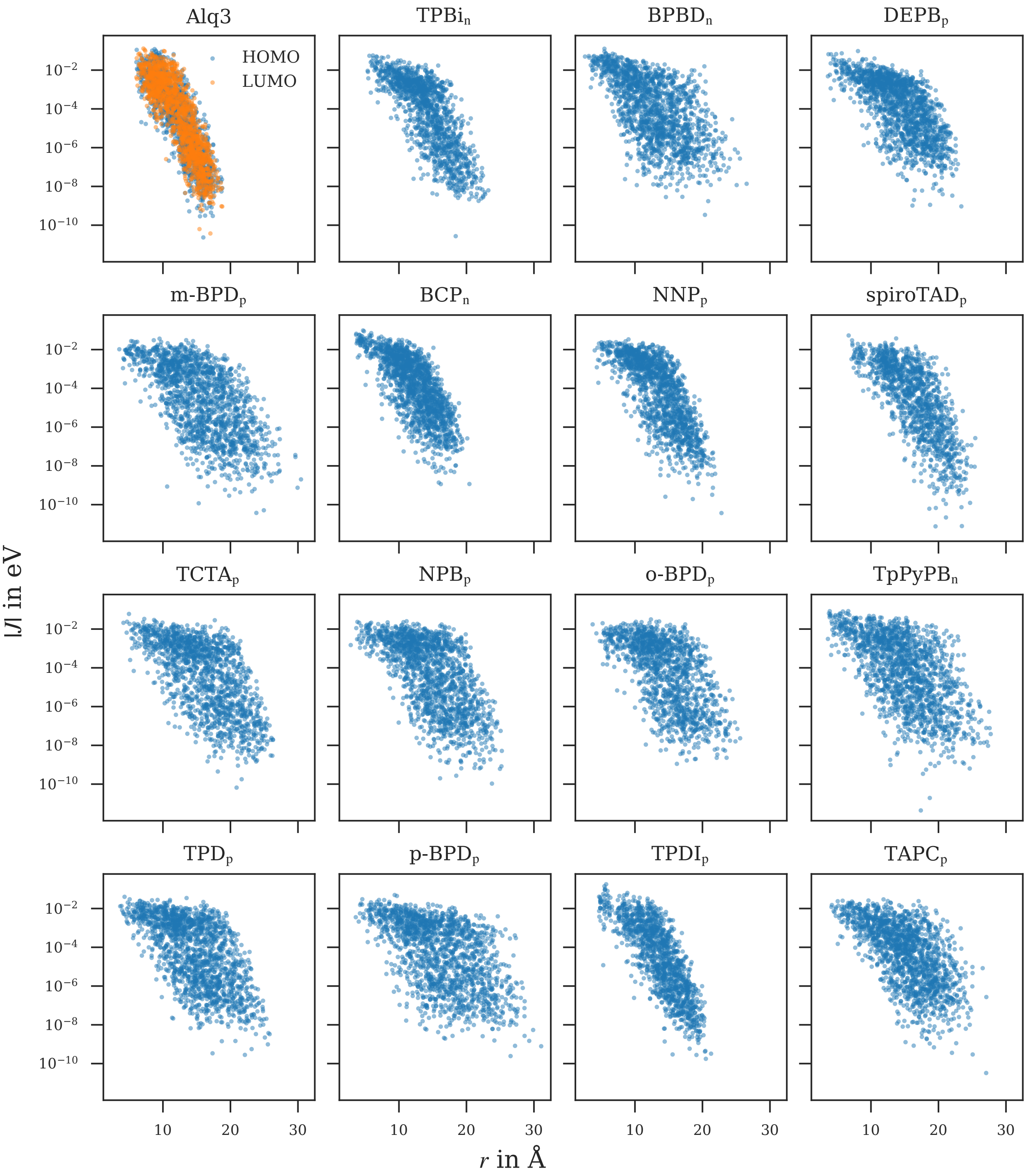

Figure S2. Distribution of electronic couplings computed with the QuantumPatch method (Friederich et al. (2014)) for all molecules studied in this work.

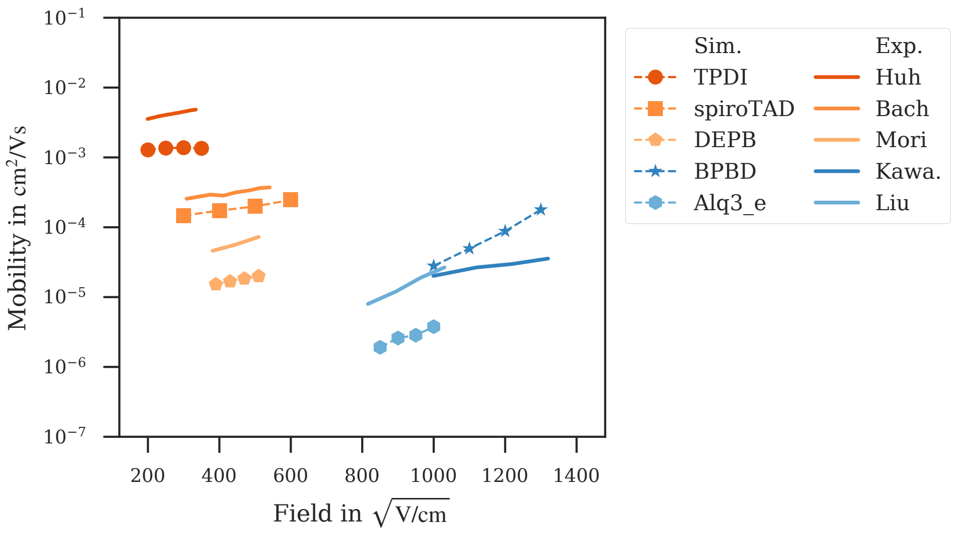

Figure S3. Computed field-dependent mobilities of the materials not shown in fig. 4 or 6 compared to experimental data reported in literature. Hole mobilities (orange) of TPDI (Huh et al. (2013)), spiroTAD (Bach et al. (2000)), DEPB (Mori et al. (1993)) and electron mobilities (blue) of TpPyPB (Su et al. (2008)), BPBD (Kawabe and Abe (2002)) and Alq3 (Liu et al. (2010)). Simulation errors are of the order of symbol size.

REFERENCES

Aydin, G. and Yavuz, I. (2021). Intrinsic Static/Dynamic Energetic Disorders of Amorphous Organic Semiconductors: Microscopic Simulations and Device Study. *J. Phys. Chem. C* 125, 6862–6869. doi:10.1021/acs.jpcc.0c11219

Bach, U., Cloedt, K. D., Spreitzer, H., and Grätzel, M. (2000). Characterization of Hole Transport in a New Class of Spiro-Linked Oligotriphenylamine Compounds. *Adv. Mater.* 12, 1060–1063. doi:10.1002/1521-4095(200007)12:14⟨1060::AID-ADMA1060⟩3.0.CO;2-R

Evans, D. R., Kwak, H. S., Giesen, D. J., Goldberg, A., Halls, M. D., and Oh-e, M. (2016). Estimation of charge carrier mobility in amorphous organic materials using percolation corrected random-walk model. *Org. Electron.* 29, 50–56. doi:10.1016/j.orgel.2015.11.021

Friederich, P., Meded, V., Poschlad, A., Neumann, T., Rodin, V., Stehr, V., et al. (2016). Molecular Origin of the Charge Carrier Mobility in Small Molecule Organic Semiconductors. *Adv. Funct. Mater.* 26, 5757–5763. doi:10.1002/adfm.201601807

Friederich, P., Symalla, F., Meded, V., Neumann, T., and Wenzel, W. (2014). Ab Initio Treatment of Disorder Effects in Amorphous Organic Materials: Toward Parameter Free Materials Simulation. *J. Chem. Theory Comput.* 10, 3720–3725. doi:10.1021/ct500418f

Fuchs, A., Steinbrecher, T., S. Mommer, M., Nagata, Y., Elstner, M., and Lennartz, C. (2012). Molecular origin of differences in hole and electron mobility in amorphous Alq 3 —a multiscale simulation study. *Phys. Chem. Chem. Phys.* 14, 4259–4270. doi:10.1039/C2CP23489K

Huh, D. H., Kim, G. W., Kim, G. H., Kulshreshtha, C., and Kwon, J. H. (2013). High hole mobility hole transport material for organic light-emitting devices. *Synth. Met.* 180, 79–84. doi:10.1016/j.synthmet.2013.07.021

Kawabe, Y. and Abe, J. (2002). Electron mobility measurement using exciplex-type organic light-emitting diodes. *Appl. Phys. Lett.* 81, 493–495. doi:10.1063/1.1494105

Kordt, P., van der Holst, J. J. M., Helwi, M. A., Kowalsky, W., May, F., Badinski, A., et al. (2015). Modeling of Organic Light Emitting Diodes: From Molecular to Device Properties. *Adv. Funct. Mater.* 25, 1955–1971. doi:10.1002/adfm.201403004

Kotadiya, N. B., Mondal, A., Xiong, S., Blom, P. W. M., Andrienko, D., and Wetzelaer, G.-J. A. H. (2018). Rigorous Characterization and Predictive Modeling of Hole Transport in Amorphous Organic Semiconductors. *Adv. Electron. Mater.* 4, 1800366. doi:10.1002/aelm.201800366

Liu, S.-W., Lee, C.-C., Lin, C.-F., Huang, J.-C., Chen, C.-T., and Lee, J.-H. (2010). 4-Hydroxy-8-methyl-1,5-naphthyridine aluminium chelate: A morphologically stable and efficient exciton-blocking material for organic photovoltaics with prolonged lifetime. *J. Mater. Chem.* 20, 7800–7806. doi:10.1039/C0JM01049A

Massé, A., Friederich, P., Symalla, F., Liu, F., Nitsche, R., Coehoorn, R., et al. (2016). Ab initio charge-carrier mobility model for amorphous molecular semiconductors. *Phys. Rev. B* 93, 195209. doi:10.1103/PhysRevB.93.195209

Mori, T., Sugimura, E., and Mizutani, T. (1993). Estimate of hole mobilities of some organic photoconducting materials using the time-of-flight method. *J. Phys. D: Appl. Phys.* 26, 452–455. doi:10.1088/0022-3727/26/3/017

Neumann, T., Danilov, D., Lennartz, C., and Wenzel, W. (2013). Modeling disordered morphologies in organic semiconductors. *J. Comput. Chem.* 34, 2716–2725. doi:10.1002/jcc.23445

Su, S.-J., Chiba, T., Takeda, T., and Kido, J. (2008). Pyridine-Containing Triphenylbenzene Derivatives with High Electron Mobility for Highly Efficient Phosphorescent OLEDs. *Adv. Mater.* 20, 2125–2130. doi:10.1002/adma.200701730
